# Imiquimod inhibits growth and induces differentiation of myeloid leukemia cell lines

**DOI:** 10.1186/s12935-018-0515-1

**Published:** 2018-01-30

**Authors:** Eva Villamón, Javier González-Fernández, Esperanza Such, José Vicente Cervera, Daniel Gozalbo, M. Luisa Gil

**Affiliations:** 1 Department of Hematology, University Hospital La Fe, Valencia, Spain; 2 Departamento de Microbiología y Ecología, Universitat de València, Burjasot, Spain; 3 Estructura de Recerca Interdisciplinar en Biotecnologia i Biomedicina (ERI BIOTECMED), Universitat de València, Burjasot, Spain

**Keywords:** Myeloid leukemia cell lines, Imiquimod, Toll-like receptor

## Abstract

**Background:**

The antitumoral effects of different Toll-like receptor (TLRs) agonists is mediated by activating immune responses to suppress tumors growth, although TLR ligands may also have a direct effect on tumoral cells. Given that TLR signaling induces hematopoietic cell differentiations this may serve as a novel differentiation therapeutic approach for AML.

**Methods:**

We investigated the effects of agonists for the ten human TLRs on the proliferation, apoptosis, cell cycle and differentiation of ten different types of myeloid leukemia cell lines (HL-60, U-937, KG-1, KG-1a, K-562, Kasumi-1, EOL-1, NB4, MOLM-13 and HEL). Proliferation was measured using the CellTiter 96^®^ Aqueous One Solution Cell Proliferation Assay (Promega). Staining and analysis with a flow cytometer was used to identify cell cycle progression and apoptosis. Differentiation was measured by staining cells with the EuroFlow™ antibody panel for AML and analyzed by flow cytometry. FlowJo software was used to analyze the cytometric data. In all experiments, statistical significance was determined by a two-tailed *t* test.

**Results:**

The activation of particular TLRs on some cell lines can induce growth inhibition and Imiquimod (a TLR 7 agonist) was the most effective agonist in all leukemic cell lines examined. Imiquimod was able to induce apoptosis, as well as to induce cell cycle alteration and upregulation of myeloid differentiation markers on some of the cell lines tested.

**Conclusions:**

Our results, together with the known efficacy of Imiquimod against many tumor entities, suggest that Imiquimod can be a potential alternative therapy to AML. This drug has a direct cytotoxic effect on leukemic cells, has the potential to induce differentiation, and can also stimulate the activation of cellular immune responses anti-AML.

## Background

Acute myeloid leukemia (AML) is a heterogeneous clonal disorder of hematopoietic progenitor cells and the most common acute leukemia in adults. AML arise from the acquisition and accumulation of genetic and/or epigenetic lesions by progenitor hematopoietic cells which results in disruption of differentiation and increase proliferation, leading to blast-cell accumulation in the bone marrow and other tissues. Both prognosis and treatment choice for AML patients are determined by several aspects, as the presence or absence of specific genetic alterations, which determine AML classification in different risk based-categories. The majority of patients with AML have an intermediate cytogenetic risk; some of these patients do well with chemotherapeutic consolidation, but others have a very poor outcome and will suffer from relapse. Generally the clinical outcome remains problematic, with a poor prognosis and an overall survival rate of only 23.6% at 5 years. The treatment of AML patients has no changed in the last years and novel agents are necessary to improve cure rates and decrease toxicity. Currently, the standard therapy approach for AML is chemotherapy using a combination of cytotoxic agents, aimed at killing highly proliferating cells. Use of differentiation therapy is a very interesting possibility allowing more efficacious and less toxic therapeutic regimen. An example is the treatment of acute promyelocytic leukemia (5–10% of AML) with all-trans retinoic acid (ATRA) as differentiation agent, that lead to the long term survival and presumed cure of well over 85% of patients. However, ATRA does not work for other types of AML, so alternative differentiation therapy need to be identified [1, 2].

Toll-like receptors (TLRs) constitute a family of pattern-recognition receptors (PRRs) that recognize molecular signatures of microbial pathogens and trigger anti-microbial and inflammatory responses. The role of TLRs on mature myeloid cells in triggering innate immune response as well as the subsequent development of adaptive immune response to microbial pathogens is well establish; however different reports have shown that functional TLRs are also expressed on hematopoietic stem and progenitor cells (HSPCs) and therefore may play a role in hematopoiesis during infection [3]. Nagai et al. [4] demonstrated that murine hematopoietic stem cells (HSCs) and their progeny express TLRs and upon in vitro exposure to soluble TLR2 and TLR4 ligands these cells are stimulated to enter cell cycle and acquire lineage markers. In addition, signaling through TLR7/8 induces the differentiation of human bone marrow CD34^+^ progenitor cells along the myeloid linage [5] and the TLR1/2 agonist Pam_3_CSK_4_ instructs commitment of human HSCs to a myeloid cell fate [6].

Given that TLR signaling induces hematopoietic cell differentiation, this could be one mechanism to explain the spontaneous remission of hematologic malignancies that have been reported associated with infection [7], and therefore serve as a novel therapeutic approach for leukemia. In this context, TLRs are expressed in different AML cell lines [8] and in mononuclear cells from the bone marrow of AML patients [9]. Moreover, Ignatz-Hoover et al. [10] showed that TLR7/8 agonist R848 promotes AML differentiation in a TLR8/Myd88/p38 dependent manner. Recently, it has been shown that S100A9, an endogenous alarmin abundantly and constitutively expressed by myeloid cells, promotes myelomonocytic and monocytic AML differentiation by activating TLR4 downstream signaling pathways [11].

Based on these previous data, the aim of the current study was to investigate the effects of agonists for the ten human TLRs on the proliferation and differentiation of different types of myeloid leukemia cell lines. We show that the activation of particular TLRs on some cell lines can induce growth inhibition and that Imiquimod was the most effective agonist in all leukemic cell lines examined. Imiquimod was able to induce apoptosis, as well as cell cycle arrest and upregulation of myeloid differentiation markers on some of the cell lines tested. Our results together with the known efficacy of Imiquimod against many tumor entities [12] suggest that Imiquimod can be a potential alternative therapeutic to AML, warranting further investigations in freshly isolated leukaemic cells.

## Materials and methods

### Human acute leukemia cell lines and culture medium

Human leukemia cell lines HL-60, U-937, KG-1, KG-1a, K-562, Kasumi-1, EOL-1, NB4, MOLM-13 and HEL were maintained in RPMI medium (Gibco) supplemented with 10% heat-inactivated fetal bovine serum (20% in the Kasumi-1 cells), 100 units/ml penicillin and 100 mg/ml streptomycin. Cells were grown at 37 °C in a humidified atmosphere containing 5% CO_2_. All cell lines used in this work were purchased from Leibnitz Institute DSMZ-German Collection of Microorganisms and Cell Cultures.

### Total RNA preparation and RT-PCR

Total RNA was prepared using the RNeasy Mini Kit (Qiagen) according to the manufacturer’s protocol. The concentration and purity of the RNA was assessed using a Nanodrop 1000 Spectrophotometer (NanoDrop Technologies). Samples with an A260/A280 ratio greater than 1.8 were selected and RNA was reverse transcribed to cDNA using the Superscript II Reverse Transcriptase (Invitrogen) using random hexamers. The cDNA generated was amplified by using primers for *ABL* as a positive control gene as previously described [13]. The Human TLR1–10 RT-Primer Set (Invivogen) was used to determine the mRNA expression pattern of human TLRs following the protocol recommended by the manufacturer. The generated PCR products were analyzed in the automated system QIAxcel Advanced System (Qiagen).

### Reagents

The TLR ligands employed in this study were purchased from Invivogen: Pam_3_CSK_4_ (a synthetic tripalmitoylated lipopeptide that mimicks the acylated amino terminus of bacterial lipoproteins, a TLR1/2 agonist used at 1 µg/ml); HKLM (heat-killed preparation of *Listeria monocytogenes*, a facultative intracellular Gram-positive bacterium, a TLR2 agonist used at 10^8^ cells/ml); Poly (I:C) (Polyinosinic-polycytidylic acid is a synthetic analog of double stranded RNA, a TLR3 agonist used at 10 µg/ml); Poly (I:C) low molecular weight (TLR3 agonist used at 10 µg/ml); LPS (lipopolysaccharide from *Escherichia coli* K12, a TLR4 agonist used at 0.5 µg/ml); Flagellin (Flagellin from *Salmonella typhimurium*, a TLR5 agonist used at 5 µg/ml); FSL-1 (a synthetic dipalmitoylated lipopeptide derived from *Mycoplasma salivarium*, a TLR6/2 agonist used at 1 µg/ml); Imiquimod (an imidazoquinoline amine analog to guanosine, a TLR7 agonist used at 10 µg/ml); R848 (an imidazoquinoline compound, a TLR7/8 agonist used at 25 µg/ml); TL8-506 (a benzoazepine compound, analog of VTX-2337, a TLR8 agonist used at 100 ng/ml); ODN2006 (synthetic oligonucleotides that contain unmethylated CpG dinucleotides, a TLR9 agonist used at 10 µg/ml). All the agonists were prepared as stock solutions in water except R848, which was prepared as a stock solution in DMSO (dimethyl sulfoxide), and added to the cultures at the indicated concentration. The control cultures for R848 treated cells were added with the appropriate amount of DMSO.

### Proliferation assay

Cells were seeded in 96-well plates (10,000 cells/well in 100 µl medium) and treated with TLR agonists for 48 or 72 h. Cells were analyzed for proliferation by a colorimetric method for determining the number of viable cells, using the CellTiter 96^®^ Aqueous One Solution Cell Proliferation Assay (Promega) as recommended by the manufacturer. The absorbance values were recorded at 450 nm after incubation at 37 °C for 3 h and corrected by subtracting the background absorbance (culture media alone). All samples were run in triplicate. Cell viability percentages were calculated as follows: cell viability % = [absorbance of treated cultures/absorbance of control cultures] × 100.

### Apoptosis and cell cycle assays

7.5 × 10^4^ cells in 100 µl medium were cultured in 96-well plates for 24, 48 or 72 h in the presence or absence of the TLR ligands. Apoptosis was evaluated by measuring annexin-V and propidium iodide (PI) binding using Annexin-V apoptosis detection kit (Santa Cruz Biotechnology). Viable cells were negative for both annexin-V and PI, early apoptotic cells were positive for annexin-V staining, and late apoptotic cells were positive for both annexin-V and PI staining. Cell cycle analysis was performed using DNA-Prep kit (Beckman Coulter) following the protocol recommended by the manufacturer for cell permeabilization and PI staining. The samples were analyzed by flow cytometry using a FACSCanto II flow cytometer (Becton–Dickinson). Samples were run in duplicate with 10,000 events counted per sample.

### Cell differentiation assay

20,000 cells in 100 µl medium were cultured in 96-well plates for 72 h in the presence or absence of the TLR agonists. Immunophenotyping was performed by staining cells with the EuroFlow™ antibody panel for AML [14], and analyzed in a FACSVerse flow cytometer (Becton–Dickinson). Data were analyzed with FlowJo 10 software.

### Statistical analysis

Statistical differences were determined using two-tailed Student’s *t* test for dual comparisons. Data are expressed as mean ± standard deviation. Significance was accepted at *P < 0.05 and **P < 0.01 levels.

## Results

### TLR mRNAs are expressed by different type of leukemia cell lines

The aim of the current study was to investigate the effects of agonists for the ten human TLRs on the proliferation and differentiation of myeloid leukemia cell lines. To address this question 10 different myeloid leukemia cell lines were used. HL-60 and Kasumi-1 (which are AML of M2 subtype), MOLM-13 (AML of M5a subtype), U-937 (a lymphoblast expressing monocytic like characteristics), K-562 (established from chronic myelogenous leukemia in terminal blast crisis), EOL-1 (from acute eosinophilic leukemia), HEL (an erythroleukemia cell line), KG-1 and the subline KG-1a (established from an erythroleukemia that evolved into AML) and NB4 (from acute promyelocytic leukemia M3).

**Fig. 1:**
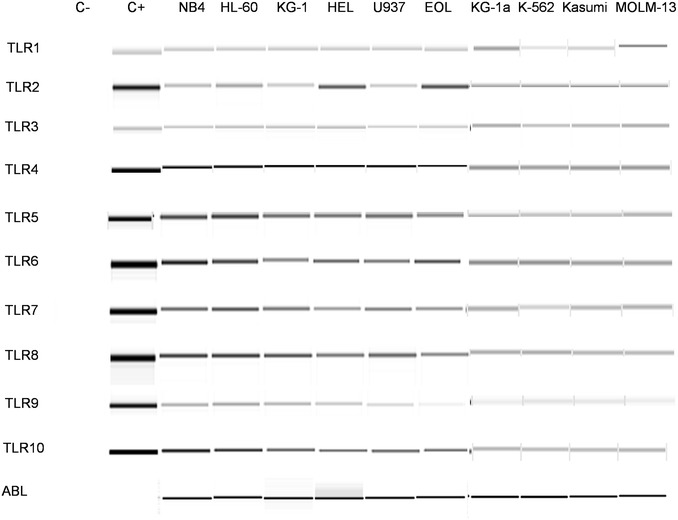
TLR mRNA expression in various types of leukemic cell lines. Analysis of gene expression of TLR1–10 in 10 different cell lines was analyzed by RT–PCR. A collection of TLR1–10 primers was provided by Invivogen, and *ABL* was used as housekeeping control gene. Positive controls for PCR were double stranded DNA provided by Invivogen, and negative controls were performed with DNA-free samples

First, the mRNA expression of TLRs in the 10 cell lines was examined by RT-PCR (Fig. 1). Results showed that all TLRs were expressed, at different levels, in all leukemic cell lines examined. This result prompted us to investigate the functional significance of this TLR expression by evaluating the effects of their respective ligands on the proliferation and differentiation of the cell lines.

### TLR7/8 agonists inhibit cell proliferation

**Fig. 2:**
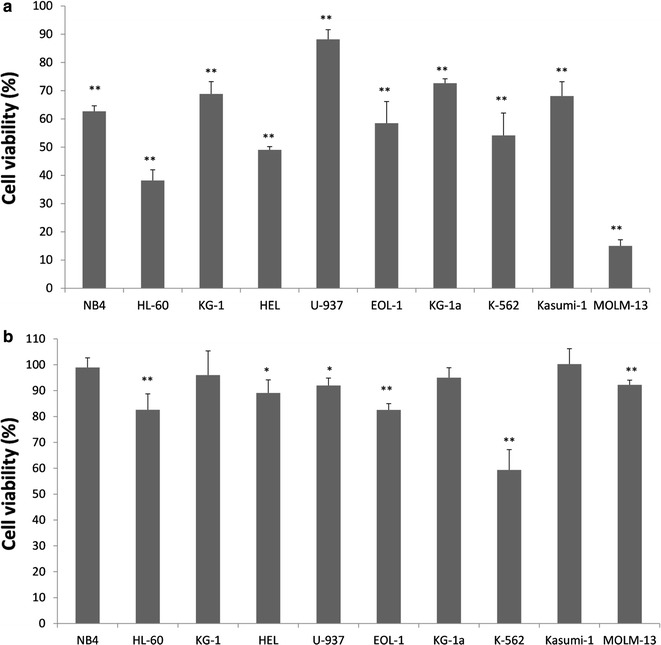
Imiquimod and R848 induce growth inhibition in various types of leukemic cell lines. The indicated leukemic cell lines were treated with Imiquimod at 10 µg/ml (**a**) or R848 at 25 µg/ml (**b**) for 72 h. Control cultures for each cell line were performed in the absence of TLR ligands. Cell growth, expressed as the percentage of cell viability relative to the control cultures (100%) for each cell line, was assessed in duplicate samples. Results are expressed as mean ± SD of pooled data from two experiments. *P < 0.05, and **P < 0.01 with respect to control untreated cells

To study the effect of the TLR ligands on the proliferation of the cell lines, we measured viable cells in the cultures incubated for 48 or 72 h, in the presence or absence of each ligand. Results showed that Imiquimod (a TLR7 ligand) treatment induced growth inhibition in all cell lines in a time-dependent manner, reaching the higher reduction in cell number at 72 h (Fig. 2a). Similarly, R848 (TLR7/8 agonist) also inhibited cell proliferation, but only in six of the ten cell lines tested, and into a lesser extent than Imiquimod (Fig. 2b). Moreover, ODN (a TLR9 ligand), was able to inhibit in a statistically significant manner the proliferation of KG-1 cells (81% cell viability at 72 h), and EOL cells (82.5% viability at 72 h). NB4 cell line was also sensitive to TLR2, TLR3 and TLR4 ligands, as the cell viability at 72 h was 85.7%, in the presence of Pam_3_CSK_4_, 86.5% in the presence of Poly (I:C), 87.5% in the presence of Poly (I:C) low molecular weight, and 84% in the presence of LPS. These differences were statistically significant in all cases.

Overall these results indicate that the activation of particular TLRs on some cell lines can induce growth inhibition, and that Imiquimod was the most effective agonist in all leukemic cell lines examined. In addition, the response to TLR challenge is dependent on the particular cell line.

### Imiquimod induces apoptosis and may also alter cell cycle

**Fig. 3:**
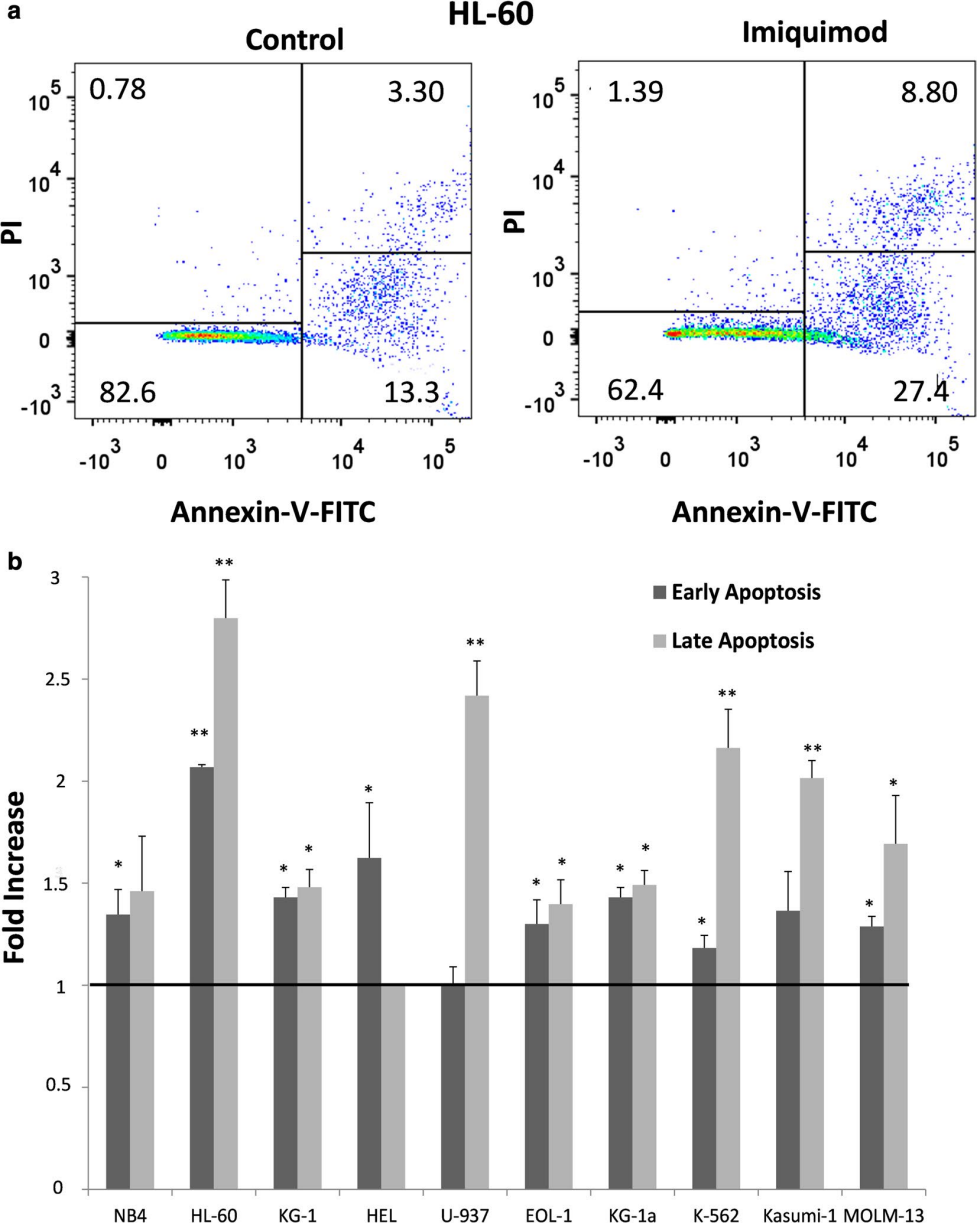
Imiquimod induces apoptosis in various types of leukemic cell lines. **a** HL-60 cells with or without (control culture) treatment (Imiquimod at 10 µg/ml for 72 h) were stained with PI and Annexin-V-FITC and analyzed by flow cytometry. The indicated percentage refers to total analyzed cells. Early apoptotic cells were positive for annexin-V staining, and late apoptotic cells were positive for both annexin-V and PI staining. Results shown are from one representative of two independent assays. **b** Fold increase (relative to control untreated cultures of each cell line) of early and late apoptotic cells in leukemic cell lines treated and analyzed following the same schedule as indicated in (**a**) for HL-60 cells. Values higher than 1 (horizontal bar) indicate increased apoptosis in Imiquimod treated cells. Data represent mean ± SD from two experiments. *P < 0.05, and **P < 0.01 with respect to control untreated cells

**N**ext, we wonder whether induction of apoptosis could account for the reduced cell proliferation in Imiquimod treated cultures. Annexin-V and PI staining was used to measure the percentage of early and late apoptotic cells by flow cytometry (Fig. 3). The percentage of apoptotic cells increased in all cell lines treated for 72 h with Imiquimod. In most cell lines an increase in both the percentage of cells in early and late apoptosis was detected, although in HEL cells only an increase in early apoptotic cells was found, and in U-937 cells only an increase in late apoptotic cells was detected. Therefore, these findings indicate that Imiquimod may lead to apoptosis in different types of myeloid leukemia cell lines. It should be noted that the frequency of apoptotic cells in control cultures did not increase during the incubation time, as similar percentages were found at 24, 48 and 72 h (not shown), whereas Imiquimod treated cultures showed a time-dependent increase in the frequency of apoptotic cells. In this context the fold increase values in total apoptotic cells for HL-60 cell line were 1.15, 1.72 and 2.40 after 24, 48 and 72 h, respectively.

**Fig. 4:**
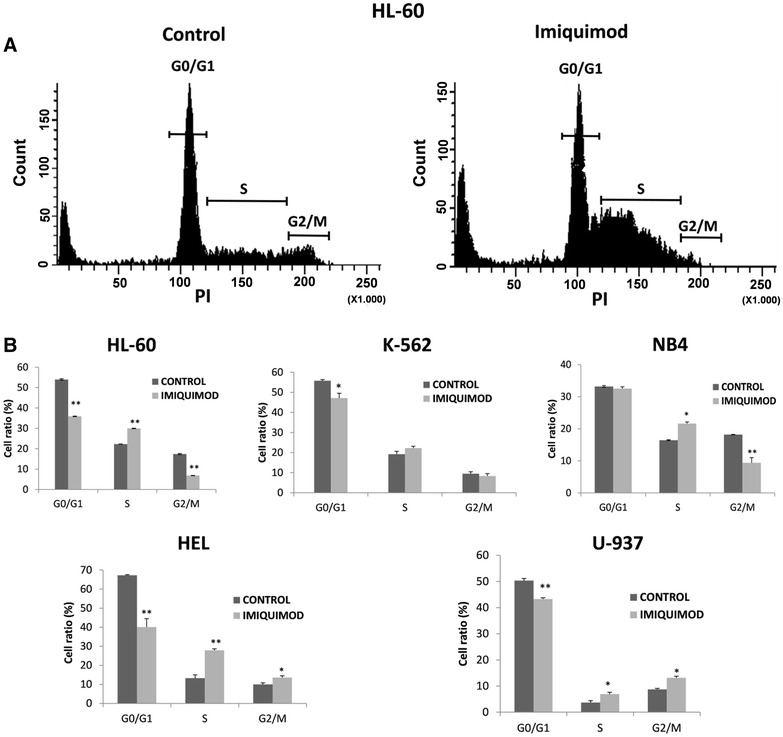
Imiquimod leads to cell cycle arrest in some leukemic cell lines. **a** HL-60 cells with or without (control culture) treatment (Imiquimod at 10 µg/ml for 72 h) were permeabilized, stained with PI, and DNA contents were analyzed by flow cytometry. Gates of cells in different phases of the cell cycle are indicated. **b** The indicated cell lines, with or without Imiquimod treatment (10 µg/ml for 72 h) were permeabilized and stained with PI, and DNA contents were analyzed by flow cytometry. The percentage of cells in each phase of the cell cycle is shown. Data represent mean ± SD from two experiments. *P < 0.05, and **P < 0.01 with respect to control untreated cells

In addition to inducing apoptosis, the inhibitory effect of Imiquimod on the growth of cell lines may also involve cell cycle arrest. To assess this possibility the proportion of cells in different phases of the cell cycle was checked in Imiquimod treated cultures (Fig. 4). Imiquimod caused alteration in cell cycle only in 5 out of 10 cell lines tested, and this effect was better observed after 72 h. The percentage of cells in the G_0_/G_1_ phase showed significant reduction at 72 h in HL-60, K-562, HEL and U-937 cells. In contrast, Imiquimod increased the cell percentage in S phase in HL-60, NB4, HEL and U-937 cells. This increase in S phase was accompanied by a decrease of cells in the G_2_/M phase in HL-60, and NB4 cells, but by an accumulation of cells in G_2_/M phase in HEL and U-937 cells. These results agree with the possibility of a generalized S phase arrest in the five cell lines and a double S and G_2_/M phases arrest in the cases of HEL and U-937 cells. However, it should be noted that in these experimental conditions Imiquimod did not cause detectable effects on cell cycle in the other five cell lines tested.

We also analyzed apoptosis and cell cycle of cells at 72 h after treatment with R848, and only statistically significant differences were found in HL-60 cell line. In these cultures R848 caused an increase in the percentage of cells in early and late apoptosis, as well as a reduction of cells in the G_0_/G_1_ and G_2_/M phases and an increase in cells in S phase (data not shown). These alterations of cell cycle and viability were similar to that induced by Imiquimod in this cell line.

Together these data suggest that cell cycle arrest contributes to the reduction of proliferation in Imiquimod treated cultures in some cell lines, while Imiquimod inhibits cell proliferation and activates apoptosis in all tested myeloid leukemia cell lines.

### Exposure of HL-60, U-937, K-562 and Kasumi-1 cells to Imiquimod induces mature myeloid marker expression

**Fig. 5:**
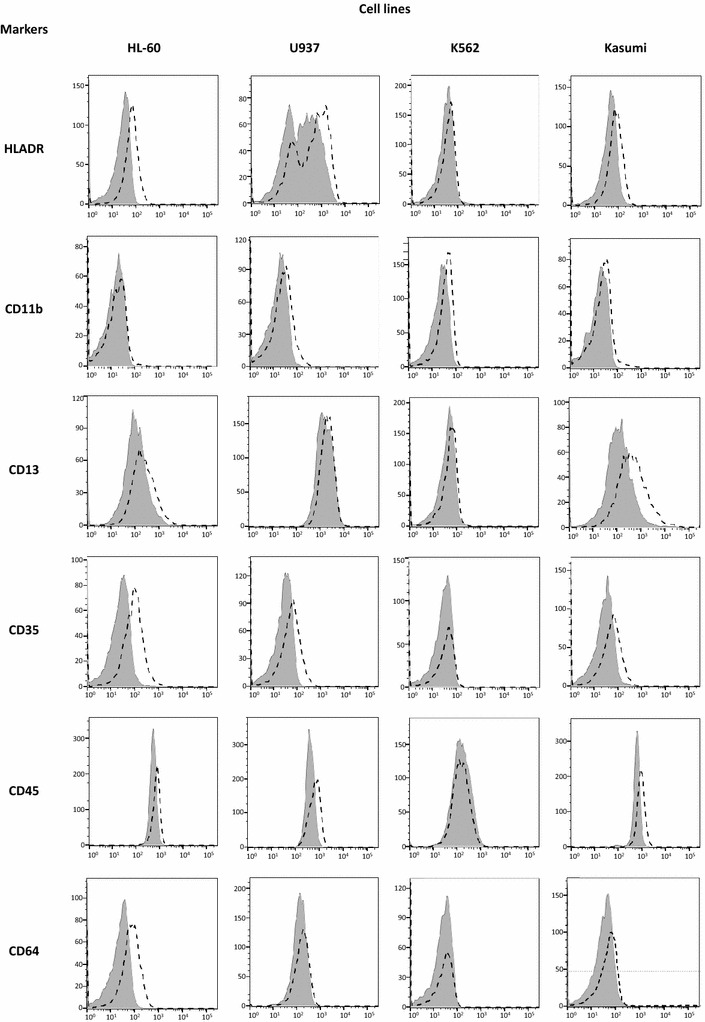
Imiquimod upregulates expression of myeloid mature markers in some leukemic cell lines. The indicated cell lines, with or without Imiquimod treatment (at 10 µg/ml for 72 h) were labeled with the EuroFlow™ antibody panel for AML (HLADR, CD45, CD16, CD35, CD13, CD64, CD34, CD117, CD11b, CD33, CD10 and CD14) and analyzed by flow cytometry. Viable cells were gated based on FS and SS values and subsequently analyzed for the fluorescence mean intensity of each specific monoclonal antibody. Only upregulated markers are shown. Results shown are representative of two independent experiments. Control cell cultures (shaded area), and Imiquimod treated cells (broken lines)

To study the effect of the TLR ligands on the differentiation of the cell lines, we performed the immunophenotyping of cells incubated for 72 h in the presence or absence of each ligand, by staining cells with the EuroFlow™ antibody panel for AML (HLADR, CD45, CD16, CD35, CD13, CD64, CD34, CD117, CD11b, CD33, CD10 and CD14). The fluorescence mean intensity (FMI) of each specific monoclonal antibody staining on viable cells (manually gated based on FS and SS values) was analyzed; only some differences were found in Imiquimod treated cells (Fig. 5), and into a lesser extent in R848 treated cultures (data not shown). More specifically, Imiquimod treated HL-60, U-937, K-562 and Kasumi cells expressed higher level of some myeloid markers: HLADR, CD11b, CD13, CD35, CD45 and CD64, are upregulated in most of these cell lines.

These results suggest that among all ligands tested, TLR7 agonists may inhibit growth of AML cell lines (partially due to cell cycle blockade and apoptosis induction) as well as induce differentiation on the remaining viable cell population.

## Discussion

TLRs are crucial drug targets due to their significant biomedical impact as they can stimulate immune responses. Generally, immunotherapy based on TLR agonists is very promising for the prevention and/or treatment of several disorders including microbial infection and cancer. Many patent applications, clinical trials and drug validation assays have involved TLR agonists, either individually or in pharmaceutical combinations with other ingredients (for a review see [15]).

The antitumoral effects of different TLR agonists is mediated by activating immune responses to suppress tumors growth by different mechanisms: increasing dendritic cell maturation and presentation, activating NK or T cytotoxic cells or increasing infiltrating immune cells in the tumors. Nevertheless, we hypothesize that TLR agonists may have a direct effect on the tumoral cells in addition to their known immune activating effect. TLR ligation on healthy HSPCs induces proliferation and differentiation towards the myeloid lineage bypassing exogenous growth factors [4, 5], and therefore the concept of TLR-targeted differentiation therapy for AML deserves to be evaluated.

Therefore, we tested agonists for the 10 human TLRs on 10 different myeloid leukemia cell lines. None of the ligands used induced proliferation, but some of them were able to induce growth inhibition; Imiquimod (TLR7 ligand) was the most effective agonist in all leukemic cell lines examined. R848 (TLR7/8 ligand) treatment also induce growth inhibition whereas TL8-506 (TLR8 ligand) had no effect, suggesting that the observed growth inhibition could be TLR7 dependent. The growth inhibition induced by Imiquimod seems to be at least partially caused by alteration of the cell cycle in some cell lines and by induction of apoptosis in all of them. In this context, the effect of Imiquimod in inhibiting proliferation and in inducing apoptosis, in all cell lines, was time-dependent.

Our results are in agreement with previous reports showing that Imiquimod induces tumor-selective apoptosis and cell cycle arrest of different type of tumor cells, including skin cancer, prostate cancer, and endometrial cancer cells [16–19]. Moreover, recently the Imiquimod derivative EAPB503 has shown to inhibit growth and to induce apoptosis in chronic myeloid leukemia cells [20], and in AML cells expressing mutant nucleophosmin 1 [21]. The induction of apoptosis in response to TLR7/8 ligands seems to occur only in tumoral cells, as it has been described that R848 enhances the survival of human CD34 progenitor cells [5].

Imiquimod is currently used as a topical and noninvasive treatment for superficial basal carcinoma, viral warts and other skin lesions in the clinic [15]. The major biologic anti-tumor activity of Imiquimod and R848 is thought to be mediated through agonistic activity towards TLR7, which results in a cell-mediated immune response [22]. In this context, it has been described that R848 greatly increases the immunostimulatory capacity of human AML cells [23], so that, when AML cells were cocultured with allogenic PBMC (peripheral blood mononuclear cells) in the presence of R848, AML cells were killed [24]. Moreover, Imiquimod can lead to recruitment of pDCs and their transformation into a subset of killer DCs able to directly eliminate melanoma tumor cells in the skin [25]. Moreover, Imiquimod acts at different levels, which appear to synergistically underline the profound anti-tumoral activity of the compound [12]. It has been described that Imiquimod also directly induces cancer cell death, like other chemotherapeutic drugs, although the mechanism of Imiquimod-induced cell death is still unclear, particularly in skin cancer cells that do not express TLR7 [16, 19]. Therefore, the effect of Imiquimod in AML cell lines may involve TLR7 dependent and/or TLR7 independent mechanisms.

As TLRs are able to induce HSPCs differentiation, then we tested the ability of the agonists to induce differentiation of AML cell lines by measuring the expression level of 12 markers that are currently used to the detection and classification of AMLs [14]. Not obvious changes in response to all the TLR ligands tested were found, except for Imiquimod and, into a lesser extent for R848. We found upregulation of the CD45 leucocyte marker, the CD11b common myeloid marker, the CD13 marker of neutrophil differentiation, and CD35, CD64 and HLADR markers of monocyte differentiation. This upregulation of mature myeloid markers was only detected, with some differences, in four cell lines. Our results are in line with a recent study showing that R848 induces differentiation of AML cells, and considerably impairs the growth of human AML cells in immunodeficient mice [10]; as R848 activates both TLR7 and TLR8, the authors tested other agonists specific for both TLR8 and TLR7 and they conclude that the differentiation induced by R848 is TLR8-mediated. However, in our experimental conditions, the effect of R848 seems to be mediated by TLR7, as we found a stronger response to a TLR7 agonist, whereas a TLR8 ligand has no effect. The reasons for these discrepancies are unknown, although they may arise from the use of different experimental conditions, such as ligand concentration used and methodology to study differentiation.

It should be noted that most established leukemia cell lines may acquire irreversible changes that make cells resistant to TLR ligand differentiation, therefore, the differentiation detected in some cell lines in response to Imiquimod, although slight may be considered significant, and strongly suggests that the effect of Imiquimod and other TLR agonists in freshly isolated leukemia cells deserves further studies.

## Conclusions

Our results reinforce the idea of TLR-targeted differentiation as a potential therapy for AML, and suggest that Imiquimod may be a useful treatment option, as this drug has a direct cytotoxic effect on leukemic cells, has the potential to induce differentiation, and can also stimulate the activation of cellular immune responses anti-AML. Further studies are needed to confirm this therapeutic option.

## Abbreviations

AML: acute myeloid leukemia
ATRA: all-trans retinoic acid
TLRs: Toll-like receptors
HSPCs: hematopoietic stem and progenitor cells
HSCs: hematopoietic stem cells
DMSO: dimethyl sulfoxide
PI: propidium iodide
PBMC: peripheral blood mononuclear cells
